# Vaccine programme stakeholder perspectives on a hypothetical single-dose human papillomavirus (HPV) vaccine schedule in low and middle-income countries

**DOI:** 10.1016/j.pvr.2018.10.004

**Published:** 2018-10-21

**Authors:** Katherine E. Gallagher, Helen Kelly, Naomi Cocks, Sandra Dixon, Sandra Mounier-Jack, Natasha Howard, Deborah Watson-Jones

**Affiliations:** a London School of Hygiene and Tropical Medicine, Department of Infectious Disease Epidemiology, Keppel St, London WC1E 7HT, United Kingdom; b London School of Hygiene and Tropical Medicine, Clinical Research Department, Keppel St, London WC1E 7HT, United Kingdom; c London School of Hygiene and Tropical Medicine, Department of Global Health and Development, Keppel St, London WC1E 7HT, United Kingdom; d Mwanza Intervention Trials Unit, National Institute for Medical Research, PO Box 11936, Mwanza, Tanzania

**Keywords:** EPI, Expanded Programme on Immunisation, HPV, human papillomavirus, ICC, Invasive cervical cancer, IPV, inactivated polio vaccine, KI, Key Informant, LMIC, low and middle-income countries, NITAG, National Immunisation Technical Advisory Group, SAGE, Strategic Advisory Group of Experts on Immunisation, WHO, World Health Organization, HPV vaccine/vaccination, Low and middle income countries, Schedule, Acceptability, Perspectives

## Abstract

**Background:**

The World Health Organization (WHO) recommends a 2-dose HPV vaccine schedule for girls aged 9–14 years. As randomised controlled trials assessing the immunogenicity and efficacy of a 1-dose schedule are ongoing, we interviewed immunisation programme managers and advisors in low and middle-income countries (LMIC) about a hypothetical, future reduction in the HPV vaccine schedule.

**Methods:**

We conducted semi-structured interviews with LMIC immunisation programme managers and national immunisation technical advisory group members (key informants; KIs) in 2017, recruited for their knowledge/experience in national HPV vaccine policy and provision. Data were analysed thematically.

**Results:**

We conducted 30 interviews with KIs from 18 countries. Perceived advantages of a 1-dose schedule included reduced logistical and financial resources needed for vaccine delivery, fewer cold chain requirements and easier integration into routine immunisation services. Perceived challenges included health worker hesitancy, resources needed to re-mobilise communities and re-train health workers, potential misrepresentation of schedule changes by anti-vaccine groups or the media. Half of interviewees suggested a WHO recommendation would be necessary prior to policy change.

**Conclusions:**

We found wide-ranging support among LMIC immunisation managers and advisors for a 1-dose vaccine schedule if research demonstrated immunological and clinical evidence of efficacy, and WHO provided a formal recommendation.

## 1 Background

Invasive cervical cancer (ICC), caused by persistent infection with human papillomavirus (HPV), is a major public health problem, especially in low-income countries [1], [2], [3]. In countries where effective cervical screening programmes are not in place or are only available on a limited scale, women frequently present late with the disease, leading to high morbidity and mortality [3], [4]. However, there are now three licensed prophylactic HPV vaccines, a bivalent, a quadrivalent and a nonavalent vaccine, that can prevent between 70% and 90% of cervical cancer cases by protecting against persistent infection with HPV vaccine genotypes, a necessary pre-requisite for the development of cervical cancer and related cervical lesions [5].

In 2014, the Strategic Advisory Group of Experts on Immunisation (SAGE) revised recommendations for the bivalent and quadrivalent HPV vaccines from a schedule of three doses to two doses at an interval of at least 6 months for girls aged 9–14 years old [6], based on evidence of non-inferior immunogenicity [7], [8], [9], [10]. Girls aged 15 years or older and HIV seropositive girls should receive three doses as per original dosage recommendations [6].

By the end of 2016, Gavi, The Vaccine Alliance, had supported 23 low and middle-income countries (LMIC) to conduct HPV vaccine demonstration programmes. Although an increasing number of LMIC have applied for Gavi support and been approved for national scale-up in recent years, the sustained financial commitment required for vaccine delivery is a key factor in some governments’ hesitancy to initiate national HPV vaccine programmes [11]. This is especially true for countries that are becoming ineligible for financial support from Gavi. These countries must increase their co-financing commitments over time until they are responsible for purchasing the vaccine at the full Gavi price (currently US$4.50 per dose) [12].

There is some evidence that a single dose of HPV vaccine may elicit a protective immune response. A combined, post-hoc analysis of 7466 women aged 18–25 years enrolled in two trials in Costa Rica and the USA suggested equivalent efficacy of one, two and three doses of the bivalent vaccine against vaccine-type persistent infection over a median follow-up of 7 years [13], [14], [15]. A study in India among 17,729 girls aged 10–18 years participating in a clinical trial that was suspended because of events unrelated to the study reported uniformly low frequencies of cumulative incident HPV16 and 18 infections over 7 years post-vaccination in all the vaccine dose groups and high vaccine efficacy in preventing persistent HPV 16/18 infections, regardless of the number of doses received [16], [17]. A recent systematic review of evidence from post-licensure observational studies reported highest effectiveness with three HPV vaccine doses but almost half of the studies found some evidence of effectiveness with one dose [18]. There are several randomised controlled trials underway to investigate the efficacy and/or immunogenicity of a single dose HPV vaccine compared to recommended schedules in Costa Rica [clinicaltrials.gov ID: NCT03180034], Tanzania [clinicaltrials.gov ID: NCT02834637] and The Gambia [19].

As scientific evidence accrues on a single dose schedule [18], [20], [21], it is important to understand the potential policy implications of changing schedules. We approached national immunisation programme stakeholders in LMIC to 1) identify the motivators for and barriers to changing the existing HPV vaccine schedule and what further information might be needed to inform a policy change to a one dose schedule in their countries in future; 2) explore potential implications of a further schedule change on the choice of delivery strategy and the perceived cost and sustainability of the HPV vaccine programme; and 3) collate experience and general attitudes towards off-label vaccine use (defined as outside of manufacturers’ recommendations).

## 2 Methods

### 2.1 Country and participant selection

A total of 27 LMIC (World Bank 2016 classifications) with some experience of HPV vaccine delivery through a demonstration project, pilot or national programme were mapped and between one and three key informants (KIs) per country were approached for interview. Due to study timelines, a purposive selection of countries was made; countries were prioritised in the first instance if they (i) supported a National Immunisation Technical Advisory Group (NITAG) or equivalent group which critically assesses evidence to inform government policy on vaccinations and/or (ii) had existing links with the study team. The final selection of countries included in the study was based on whether the Key Informants (KI) consented to be interviewed.

KIs considered for inclusion comprised (i) members of the NITAG, (ii) Expanded Programme on Immunisation (EPI) managers and/or HPV focal points within the EPI programme, or (iii) EPI country partners and/or international bodies (e.g. WHO country office, Unicef).

### 2.2 Interview procedures

After written informed consent was obtained, interviews were conducted by phone using a semi-structured interview topic guide (Supplementary Table 1). Interviews were recorded with written informed consent. If KIs did not consent to be recorded, notes were written up directly after the interview. Due to difficulties in arranging a time for phone interviews, two interviewees responded to interview questions by email.

### 2.3 Data management and synthesis

Data from interview transcripts were extracted onto an Excel matrix by the interviewer and independently verified by a second team member. The interview transcripts and recordings were stored on secure servers only accessible by the study team. Qualitative information was synthesized according to themes linked to each of the three objectives. The anonymity of interviewees was maintained for data storage purposes. However, all but one participant indicated their consent for their job title and country of interview to be used in reports of results and other dissemination materials (referred to as ‘Country Z’). Ethical approval for this study was granted by the London School of Hygiene and Tropical Medicine Research Ethics Committee.

## 3 Results

Thirty stakeholders from 18 countries provided written informed consent for interview between August and December 2017; more than one interview was conducted in 61% of countries. Interviews were conducted with nine EPI managers, 10 NITAG members, five WHO/national immunisation managers and six other individuals (Table 1).

**Table 1:** Summary of the participating countries, HPV vaccine experience and key informants.

***Region***	**Country**	**Gavi eligibility status**^a^	**National Programme Status**^b^	**HPV vaccine experience**	**Stakeholders interviewed**
***Africa***	Ethiopia	Eligible	Soon to introduce (Gavi application successful, projected introduction 2018)	Gavi demo 2015-17	1. WHO Routine Immunization Officer
					2. National Immunisation Programme Officer
					3. National Immunisation programme Coordinator
	Kenya	Eligible	Soon to introduce (Gavi application successful, projected introduction 2019)	GAP demo 2011;	1. Economist/ Policy Advisor for the Department of Policy, Planning, and Health Care Financing
				Gavi demo 2013-17	2. National Immunization Programme Officer
	Lesotho	Eligible	Introduced 2012-16 (paused)	GAP demo(s) 2009–2011;	1. WHO Immunization Officer for Lesotho
				National 2012–2016	
	Country ‘Z’	Eligible	Unknown	Gavi demo 2014-15	1. NITAG Member
	Nigeria	Eligible	Unknown	None	1. NITAG member
					2. NITAG member
	Senegal	Eligible	Soon to introduce (Gavi application successful, projected introduction 2018)	Gavi demo 2015-17	1. EPI manager, coordinator of national immunization programme
	Uganda	Eligible	Introduced 2015	Demo(s) 2008-14	1. EPI Team Leader
				Natl. 2015-	2. EPI HPV Focal Person
	Zambia	Eligible	Soon to introduce (Gavi application successful; projected introduction 2019)	GAP demo 2013-14	1. National EPI Manager
	Zimbabwe	Eligible	Soon to introduce (Gavi application successful, projected introduction 2018)	Gavi demo 2015-17	1. Director of Epidemiology and Disease Control and Zimbabwe NITAG Chairperson
					2. EPI Manager
***South East Asia***	Lao PDR	Eligible	Soon to introduce (Gavi application successful; projected introduction 2019)	Gavi demo 2013-15	1. National immunisation Programme Manager
					2. NITAG member
	Nepal	Eligible	Unknown	Demo 2008–2015	1. Technical advisor to NITAG 2. Vaccine Officer for WHO Nepal Office
	Solomon Islands	Eligible	Soon to introduce (Gavi application successful; projected introduction 2019)	Gavi demos 2015-17	1. EPI Programme Officer
***Latin America***	Argentina^c^	Ineligible	Introduced 2011	Natl: 2011-	1. Director of Preventable Diseases (MoH)
	Bolivia	Accelerated transition phase^d^	Introduced 2017	Demos 2009–2011	1. PAHO/WHO Bolivia EPI Consultant
				Natl.: 2017-	2. President of NITAG
	Brazil	Ineligible	Introduced 2014	Demos 2010-12	1. Coordinator of National Immunization Program at MoH, NITAG member
				Natl. 2014-	
	Colombia	Ineligible	Introduced 2012	Natl. 2012-	1. NITAG and EPI National Coordinator
					2. Other
	Peru	Ineligible	Introduced 2011	Demos 2007–2010	1. Former Minister of Health; Dean of the Public Health Faculty at the Cayetano Heredia in Peru
				Natl. 2011-	2. NITAG President
***Eastern Europe***	Moldova	Fully self-financing	Unknown	GAP Demo 2010-11	1. Head of Epidemiology Department of preventable diseases through vaccination within the National Centre for Public Health and NITAG member
					2. NITAG Secretary

GAP: Gardasil Access Program

a Gavi-eligible: 3-year average gross national income per capita <1580 USD [12]

b The status of the country's plans for national Introduction of HPV vaccine was informed by investigator's previous contact with the country representatives and Gavi's forecasting of successful applications at the time this study was being conducted. ‘Soon to introduce’ meant an application for HPV vaccine support had been prepared by country representatives (and may have been submitted for Gavi's consideration or a date for submission was planned).

c National programme was modified in 2017 to include delivery to boys and catch up vaccination to 26 years of age for people living with HIV.

d Gavi support was provided for the first year of the national programme only.

### 3.1 Perceived advantages of a 1 dose HPV vaccine schedule

All of the 30 KIs interviewed had a good comprehension of the vaccine's effectiveness; 27 KIs (90%) representing all 18 countries thought that a future, hypothetical schedule change to a single dose HPV vaccine schedule would be supported by key stakeholders within their country. Three KIs did not respond to the question directly. KIs cited a range of potential advantages to a single dose HPV vaccine schedule, 15 KIs anticipated a reduction in the financial resources needed for vaccine delivery (Table 2).

**Table 2:** Perceived advantages of a single dose HPV vaccine schedule.

**Perceived advantages of a 1-dose schedule**	**KIs N= 30**^a^	**Countries N = 18**
Reduction in HPV vaccine programme costs	15	11
Operational/logistical advantages^b^	15	12
High coverage of one dose^c^	7	7
Easy integration into routine immunisation services/ other services e.g. annual child health days	7	6
Lower cold chain requirements	3	2
Increased community acceptability due to fewer visits/ injections	3	3
Potential to extend HPV vaccination to a wider cohort or boys or older women given the reduced cost of a one dose schedule^d^	2	2
Did not want to respond	3	3

a KIs cited more than one advantage.

b Operational or logistical advantages referred to easier implementation of one dose in schools including less interruption of school activities, fewer visits and ease of integration into routine immunisation services or existing outreach services.

c Seven representatives from seven countries reported high first dose coverage in previous HPV vaccine programmes but lower second dose coverage and would therefore welcome the opportunity to be able to report the higher first dose coverage as overall coverage.

d Brazil and Bolivian representatives mentioned the savings from a single dose programme could be reinvested to widen the target group for HPV vaccine.

The relative logistical ease of a one dose schedule, which would not require girls to be traced for their second dose, was also a common theme:

*“[A one dose schedule] will be less work for nurses.. it's less time vaccinating.. so they have more time doing other activities in the clinics…. We have a problem with logistics and transportation in the country.” KI, Solomon Islands.*

### 3.2 Perceived barriers to a 1 dose HPV vaccine schedule

KIs from the same country were not always in agreement in their perception of potential barriers to the introduction of a single dose schedule. Fourteen KIs from 13 countries did not anticipate any barriers to the hypothetical introduction of a one dose HPV vaccine schedule in their country. Eight KIs from 6 countries cited concerns around gaining community or individual acceptance of a single dose schedule in place of two/three doses. KIs referred to the potential for individuals or communities to question whether one dose would be sufficient to provide adequate protection, given the mobilisation and communication to date on the importance of receiving two or three doses (Table 3).

**Table 3:** Perceived barriers to 1 dose HPV vaccine schedule.

**Perceived barriers to 1 dose HPV vaccine schedule**	**KIs N = 30**^a^	**Countries N = 18**
No barriers perceived	14	13
Community or individual acceptance^b^	8	6
Acceptability among healthcare workers	6	6
Negative media or anti-vaccine groups	3	3
Cost of re-mobilisation/ retraining necessary	5	5
Did not respond/ did not want to hypothesize	2	2

a KIs cited more than one barrier.

b Community or individual acceptance refers to communities questioning whether one-dose is sufficient, or concerns raised over whether 2/3 doses were therefore too much.

In Kenya, there was concern that a further schedule change to one dose soon after the schedule was reduced from three to two doses, could be perceived by communities as a lack of sufficient evidence about which schedule is effective:

*"It does raise concerns of very fast changes. i.e. it implies that comprehensive research was not really done at the outset and we are more on a trial and error phase which many would not be comfortable with." KI, Kenya.*

Seven KIs from six countries cited potential acceptability issues among HCWs as a potential barrier to a one dose HPV vaccine programme, given that their training to date has specified two/three doses. Five KIs stated that sourcing the resources needed for the retraining of HCWs and remobilisation of the community would be a challenge if no extra support was provided (Kenya, Ethiopia, Bolivia, Uganda, Country Z). One KI explained that a formal recommendation from the WHO would be useful to dispel any resistance propagated by anti-vaccine groups. KIs from three countries mentioned the potential for information around a further schedule change to be used negatively by girls who had already been vaccinated and who were affected by adverse events following immunisation or by anti-vaccine groups, or by the media (Colombia, Peru, Kenya):

*“I think [a one dose schedule] could be very good for the ones who are not vaccinated yet, but … the ones who are saying that they suffered adverse effects from the vaccine.. they could say “you gave us more doses than we needed and that is why we got ill”.” KI, Colombia*

*“I always fear the anti-vaccine groups for example, if in some years ahead someone who received only one dose developed cancer” KI, Peru*

Another disadvantage cited by one KI (Ethiopia) included that a single visit would impact the frequency of contact between the HCW and the adolescent, thereby decreasing the opportunity for the provision of sexual and reproductive health (SRH) services for adolescents.

### 3.3 Information needs for a future schedule change

All KIs from all 18 countries stated at least one source of information that they perceived would be required for the decision-making process around a future, hypothetical HPV vaccine schedule change (Table 4). Often multiple sources of information were listed.

**Table 4:** Countries classified by the highest level of evidence that KIs perceived may be needed for any future discussions on a further reduction of the HPV vaccine schedule.

**Reported evidence needed for consideration**	**Immunogenicity data**	**Efficacy data against a clinical endpoint (+/- immunogenicity data)**	**WHO position paper (+/- efficacy +/- immunogenicity data)**
**Countries**	Nigeria	Lesotho	Ethiopia
	Colombia	Country ‘Z’	Senegal
	Moldova	Argentina	Uganda
		Brazil	Laos
		Zambia	Nepal
		Zimbabwe	Solomon Islands
			Bolivia
			Peru
			Kenya

KIs from nine countries thought that a WHO position paper or a WHO recommendation would be required before policy makers would consider a change to a one dose HPV schedule. Only KIs from Argentina stated explicitly that policy makers would not necessarily wait for formal WHO recommendations to enact a change in vaccination policy (e.g. schedule changes), unless there was significant controversy around the decision. KIs from eight countries (Argentina, Brazil, Lesotho, Peru, Senegal, Uganda, Country Z and Zambia) stated that evidence on the efficacy of one dose of HPV vaccine against HPV infection and/or clinical endpoints compared to two or three doses would be important in the decision-making process around recommending a change in schedule. KIs from 10 countries (Lesotho, Country Z, Nigeria, Senegal, Nepal, Argentina, Bolivia, Brazil, Colombia and Moldova) mentioned that evidence on the immunogenicity of one dose of HPV vaccine compared to two or three doses would be important for the decision-making process.

### 3.4 Perceived implications of a schedule change on HPV vaccine delivery strategy and the affordability

KIs from all 18 countries discussed the perceived implications of a schedule change on the vaccine delivery strategy. The majority of KIs stated that a change in schedule would not alter the recommended delivery strategy for HPV vaccine in their country (Table 5). KIs from just two countries (Country Z and Zambia) mentioned that a single dose strategy may be easier to integrate with existing annual health day campaigns rather than delivery through their routine existing health system activities and therefore a change of delivery strategy may be considered.

**Table 5:** HPV vaccine delivery experience and perceived implications of a further schedule change to the vaccine delivery strategy.

Country	HPV vaccine experience	Delivery strategy experience	KI perspective on potential changes to delivery strategy if 1 dose schedule was implemented
Ethiopia	Gavi demo 2015–17	Demo exp: School based + community outreach	No change
		Natl. plans: School based	
Kenya	GAP demo 2011;	Natl. plans: School based	No change but would propose to integrate with another service delivered in schools such as deworming or health education on hygiene i.e the school health days or malezi bora campaigns
	Gavi demo 2013–17		
Lesotho	GAP demo(s) 2009–2011;	Natl. exp: School-based	Integrate into routine immunisations services at the health facility with outreach
	National 2012-		
Country ‘Z′	Gavi demo 2014–15	Demo exp: School + health centre based + outreach	Uncertain; potentially integrated with annual vitamin A campaigns
Nigeria	None	N/A	Uncertain
Senegal	Gavi demo 2015–17	Demo exp/ Natl. plans: School + health centre based + outreach	No change
Uganda	Demo(s) 2008–14	Natl. exp: health facility based + outreach	No change
	Natl. 2015-		
Zambia	GAP demo 2013–14	Demo exp: Schools + health facilities	Potentially integrate into Child Health Week campaign, which includes deworming and immunisation
Zimbabwe	Gavi demo 2015–17	Demo exp: School + health centre based + outreach.	No change
		Natl. plans: School + health facility	
Lao PDR	Gavi demo 2013–15	Demo exp: School + health centre based + outreach.	Unknown
Nepal	Demo 2008–2015	Demo exp: School + health centre based	No change
Solomon Islands	Gavi demos 2015–17	Demo exp: School + health centre based + outreach.	No change; easier to integrate with TT and Oral Polio vaccine outreach delivered by the school health programme
Argentina	Natl: 2011-	Natl. exp: school + Health facility + outreach (dependent on province)	No change; currently integration of Hepatitis B, rubella, meningitis and first dose of HPV
Bolivia	Demos 2009–2011	School based	No change; possibly integrate with tetanus
	Natl: 2017-		
Brazil	Demos 2010–12	Natl. exp: school + Health facility	No change. Integrated with Meningitis C and diphtheria vaccine
	Natl. 2014-		
Colombia	Natl. 2012-	Health centres	No change
Peru	Demos 2007–2010	Natl. Exp: School based	No change
	Natl. 2011-		
Moldova	Gap Demo 2010–11	Demo. Exp: School-based	Uncertain

Abbreviations: Demo: Demonstration project; exp: experience; Natl: National programme. Information collated from KIs and Gavi application documents at gavi.org.

KIs from 15 countries offered their opinions on affordability and KIs from the same country were in agreement on this point. Four KIs from 3 countries were unsure as to what extent the reduction of schedule would affect the cost of the HPV vaccination programme. The remaining 18 KIs from 12 countries (Argentina, Bolivia, Brazil, Ethiopia, Kenya, Lao, Lesotho, Moldova, Country Z, Nepal, Zambia, Zimbabwe) perceived that a reduction in schedule would reduce the overall cost of vaccine procurement and delivery. Lesotho mentioned that a reduction in schedule and concomitant reduction in programme cost could help re-start the national HPV vaccination programme. The KI from Moldova stressed that despite a potential reduction in cost, political will would need to increase for the HPV vaccine to be successfully introduced nationally:

*“There is not sufficient political support at this moment… Currently the National Immunization Program is entirely covered from the State budget and HPV vaccine is the most expensive one out of the whole spectrum of vaccines proposed for funding by the Immunization Program” KI Moldova*

Despite recognising that a reduced schedule could potentially substantially reduce the cost of the HPV vaccine programme, KIs from 7 countries (Ethiopia, Lao, Country Z, Nepal, Solomon Islands, Uganda, Zimbabwe) mentioned residual concerns over the sustainability of their HPV vaccine programme:

*“It is already hard to sustain the vaccines that we have in our programme. Introducing a new one is increasing the burden that we already have. But of course… the one dose approach would be much, much easier to manage.” KI Country Z*

Two KIs (Ethiopia, Zimbabwe) specifically stated that for the programmes to be sustainable, the cost of the vaccine still needs to reduce, despite a one dose schedule and Gavi support.

*“[One dose] will help in the shorter-term… HPV will only be sustained longer-term if the price of that 1 dose is negotiated again” KI Ethiopia*

*“The price of the vaccine still needs to be reduced even with a reduction to one dose.” KI Zimbabwe*

Among the 12 countries eligible for Gavi support, 8 KIs in 6 countries mentioned the approaching transition away from Gavi support as a contributing factor to their assessment of the implications of a one dose schedule on cost and sustainability of the HPV programme:

*“Current national rollout support from Gavi ends in 2021. The country is going to take on board [the cost of] not just HPV vaccine, but other vaccines as well… The executives are aware of our transition, that we are graduating soon from Gavi, so they are working out whether other support will come once we have graduated from Gavi.” KI Solomon Islands*

KIs from Kenya and Uganda specifically stated that a single dose programme could alleviate costs and resources associated with tracing and catch-up of girls who miss their second/third dose.

### 3.5 Views on off-label vaccine use

KIs from nine of the 18 countries interviewed knew of no prior experience of off-label vaccine use in their country (Table 6). KIs from four countries reported that policy makers were currently considering off-label vaccination with the delivery of fractional doses of inactivated polio vaccine (IPV). KIs from all five of the Latin American countries interviewed reported that their countries had had previous experience of national introduction of vaccines off-label without/prior to WHO recommendations e.g. the use of a single dose schedule of Hepatitis A vaccine (Argentina, Brazil), a single dose schedule of varicella vaccine (Argentina), early introduction of a reduced schedule of three doses of pneumococcal conjugate vaccine (Argentina, Peru), fractional dose IPV (Colombia), prolonged storage of IPV (Bolivia) and administration of a pertussis vaccine in pregnancy (Argentina, Colombia).

**Table 6:** Summary of key findings by country.

	**Date of national HPV vaccine intro’**	**Readiness and perceived advantages**			**Barriers**			**Information needs**	
**Country**		Would support 1-dose	NITAG in place	Experience of off-label vaccine use	Community mobilisation needed^a^	HCW mobilisation needed^b^	Concerns over negative media	WHO recommendation required	Other country lessons on 1-dose
Ethiopia	2018^f^	Yes	Yes	No	Yes	Yes		Yes	Yes
Kenya	2019^f^	Yes	Yes	No	Yes	Yes	Yes	Yes	
Lesotho	2012–16	Yes	None (in development)	Under consideration					
Country Z	NA	Yes	Yes	No					
Nigeria	NA	Yes	Yes	No					
Senegal	2018^f^	Yes	Yes	No	Yes			Yes	
Uganda	2015-	Yes	Yes	No		Yes		Yes	
Zambia	2019^f^	Yes	Yes	Under consideration	Yes	Yes			
Zimbabwe	2018^f^	Yes	Yes	No^c^	Yes	Yes			
Lao PDR	2019^f^	Yes	Yes	No^c^	Yes			Yes	Yes
Nepal	NA	Yes	Yes	Under consideration				Yes	
Solomon Islands	2019^f^	Yes	None	Yes (current)				Yes	
Argentina	2011-	Yes	Yes	Yes (past)				No^d^	
Bolivia	2017-	Yes	Yes	Yes (past)				Yes^e^	
Brazil	2014-	Yes	Yes	Yes (past)					
Colombia	2012-	Yes	Yes	Yes (past)		Yes	Yes		Yes
Peru	2011-	Yes	Yes	Yes (past)			Yes	Yes	
Moldova	NA	Yes	Yes	No					

a Concerns were raised over community acceptance of (another) schedule change/ mistrust/ the additional resources needed to re-mobilise the community.

b Concerns over health care worker acceptance of a new schedule and the additional resources needed for re-training.

c KIs indicated any approval for off-label use would take a long time to be processed and/or would not be considered.

d KIs in Argentina were the only KIs to indicate that WHO recommendation would not necessarily be needed prior to introduction of a change in HPV schedule (other KIs either explicitly stated they would be needed or did not mention them).

e Bolivian KIs also mentioned manufacturer recommendations for the schedule change may be needed.

f Projected introduction date based on Gavi application; NA: unknown/ not available.

## 4 Discussion

Key immunisation stakeholders from 27 countries were approached for interview; thirty key informants from 18 countries (67%) provided written consent to be interviewed. Overall, the KIs interviewed suggested there would be support for a hypothetical future simplification of the HPV vaccine schedule to a single dose. It was generally acknowledged that this would reduce the resources required for delivery, the discomfort and inconvenience to the vaccinees and the financial commitment required for vaccine procurement. A number of KIs stressed that, although a single dose schedule might alleviate some of the logistical and financial challenges of HPV vaccine delivery, there remained a need for strong political will, social mobilisation and healthcare worker training to ensure programme success and longevity. Some KIs also called for continued efforts to reduce the vaccine per-dose cost, citing residual concerns over HPV vaccine programme sustainability. Due to these remaining concerns over the sustainability of even a single-dose programme, only two respondents saw the reduction in schedule as an opportunity to expand coverage to other target groups.

The decision-making processes and information needs for a future hypothetical one dose schedule change were fairly similar across countries. WHO recommendations on vaccine introduction and delivery play an important role, especially in Africa, in decisions to introduce or change vaccine programmes. WHO recommendations are also used to reassure communities about a change in vaccine policy should negative media or rumours arise. KIs from half the countries interviewed stated that they felt a WHO recommendation for a single dose schedule would be needed prior to a schedule change in their country, whereas others were uncertain on this point. KIs from three of the 18 countries specifically stated that they would want to hear lessons from other countries that had introduced a single dose schedule i.e. that they would not want to be the first to implement. There were concerns that the change in policy could fuel negative media coverage of the national immunisation programme.

Known prior experience of off-label vaccine use was concentrated in the South American countries that were interviewed. There may be other examples of off-label vaccine use that were not reported by the KIs. There were a number of limitations with the study; we were not able to collect information from nine of the 27 countries approached for interview and the interviews conducted represented the individual opinions of the KIs rather than a consensus reached by those making of advising vaccination policy. There may be different conclusions drawn e.g. on the information needed prior to a schedule reduction or the implications of a schedule change on the recommended delivery strategy, if the decision-making processes in the country were followed and relevant committees and advisory groups consulted.

## 5 Conclusions

In conclusion, we found wide ranging support among 27 immunisation programme stakeholders from 18 LMICs for a future further reduction in the HPV vaccine schedule to a single dose, if there is immunological and clinical evidence of efficacy. Randomised controlled trials are underway to analyse whether a single dose of HPV vaccine delivers non-inferior immunogenicity and efficacy compared to 2 and 3 dose regimens.
