# Supplementary material for: Vaccine programme stakeholder perspectives on a hypothetical single-dose human papillomavirus (HPV) vaccine schedule in low and middle-income countries
Source: Papillomavirus Res. 2018 Oct 21;6:33–40. doi: 10.1016/j.pvr.2018.10.004 (PMC6218645; doi:10.1016/j.pvr.2018.10.004)
Supplement: Supplementary file 1 — Supplementary material [file mmc1.docx]

Supplementary Table 1. Summary of key informant interview topic guide, by objective

| **Study objectives** | **Summary of interview topic guide** |
| --- | --- |
| **Objective 1:**  Motivators, barriers and information needs for a future, hypothetical HPV vaccine schedule change | What are the perceived advantages of one-dose schedule? |
|  | What are the potential barriers to a one-dose schedule? |
|  | How would a decision on any future schedule change be made and who would be involved? |
|  | What information/evidence would be needed for a future schedule change? |
|  | What other factors influence the decision to change schedule? |
|  | Would a one-dose schedule influence the country’s decision to continue/re-start/pause the HPV vaccine programme? |
| **Objective 2:**  Perceived implications of a further schedule change on the choice of delivery strategy and the perceived cost and sustainability of the programme | How might a change to a one-dose schedule influence the recommended delivery strategy for HPV vaccine? |
|  | How might a change to a 1-dose schedule influence the integration of HPV vaccine into the routine immunisation schedule? or integration with other interventions? |
|  | What might be the implications of a change to a one-dose schedule on the affordability and sustainability of the HPV vaccine programme? |
| **Objective 3:**  Experience with off- label vaccine use | What experience do you know of, in your country, of off-label vaccine use (using a vaccine outside of manufacturer recommendations)? |
|  | If there has been experience of off-label use: how was the initial decision made to deliver the vaccine outside of manufacturer recommendations? |
|  | If there has been no experience of off-label use: how might a decision be made to use a vaccine off label (i.e. who would be the key actors involved, stakeholders, advisory groups and data needs to inform decision)? |
